# The Effectiveness of Text Messaging for Detection and Management of Hypertension in Indigenous People in Canada: Protocol for a Randomized Controlled Trial

**DOI:** 10.2196/resprot.7139

**Published:** 2017-12-19

**Authors:** Karen Yeates, Norm Campbell, Marion A Maar, Nancy Perkins, Peter Liu, Jessica Sleeth, Carter Smith, Colin McAllister, Diane Hua-Stewart, George Wells, Sheldon W Tobe

**Affiliations:** ^1^ Department of Medicine Queen's University Kingston, ON Canada; ^2^ Department of Medicine University of Calgary Calgary, AB Canada; ^3^ Faculty of Medicine Northern Ontario School of Medicine Sudbury, ON Canada; ^4^ Sunnybrook Research Institute, Sunnybrook Health Sciences Centre Department of Medicine University of Toronto Toronto, ON Canada; ^5^ University of Ottawa Heart Institute Ottawa, ON Canada; ^6^ Department of Biology & Psychology SSP Queen's University Kingston, ON Canada; ^7^ Perspect Management Consulting Regina, SK Canada

**Keywords:** hypertension, health services, indigenous, chronic disease, disease management, telemedicine, text messaging

## Abstract

**Background:**

Hypertension, the leading cause of morbidity and mortality, affects more than 1 billion people and is responsible globally for 10 million deaths annually. Hypertension can be controlled on a national level; in Canada, for example, awareness, treatment, and control improved dramatically from only 16% in 1990 to 66% currently. The ongoing development, dissemination, and implementation of Hypertension Canada’s clinical practice guidelines is considered to be responsible, in part, for achieving these high levels of control and the associated improvements in cardiovascular outcomes. A gap still exists between the evidence and the implementation of hypertension guidelines in Indigenous communities in Canada, as well as in low- and middle-income countries (LMICs). The rapid rise in the ownership and use of mobile phones globally and the potential for texting (short message service, SMS) to improve health literacy and to link the health team together with the patient served as a rationale for the Dream-Global study in both Canada and Tanzania.

**Objective:**

The primary objective of the Dream-Global study is to assess the effect of innovative technologies and changes in health services delivery on blood pressure (BP) control of Indigenous people in Canada and rural Tanzanians with hypertension using SMS messages and community BP measurement through task shifting with transfer of the measures electronically to the patient and the health care team members.

**Methods:**

This prospective, randomized blinded allocation study enrolls both adults with uncontrolled hypertension (medicated or unmedicated) and those without hypertension but at high risk of developing this condition who participate in a BP screening study. Participants will be followed for at least 12 months.

**Results:**

The primary efficacy endpoint in this study will be assessed by analysis of variance. Descriptive data will be given with the mean and standard deviation for continuous data and proportions for ordinal data. Exploratory subgroup analyses will include analysis by community, sex, mobile phone ownership at baseline, and age. The knowledge gained from the text messages will be assessed using a questionnaire at study completion, and results will be compared between the groups.

**Conclusions:**

This study is expected to provide insights into the implementation of an innovative system of guidelines- and community-based treatment and follow-up for hypertension in Indigenous communities in Canada and in Tanzania, an example of an LMIC. These insights are expected to provide the information needed to plan scalable and sustainable interventions to control BP virtually anywhere in the world.

**Trial Registration:**

Clinicaltrials.gov NCT02111226; https://clinicaltrials.gov/ct2/show/NCT02111226 (Archived by WebCite at http://www.webcitation.org/6v7IdYzZh)

## Introduction

### Background and Rationale

Worldwide, hypertension is the leading cause of morbidity and mortality, affecting more than 1 billion people and responsible for 10 million deaths annually [1]. Hypertension increases the burden of disease globally through cardiovascular disease, kidney disease, and dementia [2-4]. For every 20-mm Hg increase in systolic blood pressure (BP) above 115 mm Hg, and every 10-mm Hg increase in diastolic BP above 75 mm Hg, there is a doubling in the risk of mortality from cardiovascular disease [5]. Placebo-controlled studies of BP lowering in people with hypertension demonstrate a 17% reduction in major cardiovascular events for every 5-mm Hg fall in systolic BP [6]. Hypertension can be controlled at the national level. For example, BP control improved dramatically in Canada from only 16% in 1990 to 66% at present [7,8]. The ongoing development, dissemination, and implementation of Hypertension Canada’s clinical practice guidelines is considered to be responsible to a great extent for achieving these high levels of awareness, treatment, and control, which in turn are linked to improved cardiovascular outcomes [9]. However, although BP control and treatment have improved nationally, the improvements are not distributed equally at the regional level linked to disparities in the determinants of health.

In Tanzania and other low-income countries, hypertension control presents a significant challenge because of deficiencies in primary health care resources such as health care providers and antihypertensive medications. Tanzania, like many countries in sub-Saharan Africa, is experiencing an epidemiological transition from a disease burden of mostly communicable diseases and injuries to a disease burden increasingly dominated by noncommunicable diseases such as cardiovascular disease, diabetes, and cancer [10]. The approaching wave of chronic diseases that require monitoring and access to drugs is a threat to the health care system in Tanzania. At present, in Tanzania, the prevalence of hypertension is between 35% and 40% in adults, with awareness of hypertension only 20%, treatment 10%, and control approximately 5% [11]. Many of the determinants of health affecting Canada’s Indigenous communities are shared by people in low- and middle-income countries (LMICs) [12]. Thus, interventions that work in LMICs may have a role in Indigenous communities and other similar populations.

In Canada, rates of hypertension in Indigenous populations are 20%, similar to the rest of the country [13]. Although data for treatment and control rates in Indigenous populations are lacking, higher cardiovascular disease rates suggest poorer control. A framework for discussion on how to improve the prevention, management, and control of hypertension from 2011 to 2020 describes this situation and calls for Indigenous populations to have similar rates of BP surveys and health indicators as the rest of the population [14]. Inequities in the social determinants of health—poverty, in particular—lead to poorer health outcomes in Indigenous communities than in the general Canadian population [15]. The social determinants particularly important for Indigenous health include income, education, employment, living conditions, adequate housing, and access to culturally competent health services. Indigenous health is also affected by the consequences of colonialism, including loss of language and ways of life and dispossession of traditional lands. This has led to food insecurity; environmental deprivation; and spiritual, emotional, and mental disconnectedness [16,17]. Compounding these issues are frequent experiences of social exclusion, discrimination, and racism [18,19]. All of these issues must be considered when undertaking a research project with Indigenous communities.

The DREAM3 (Diabetes Risk Evaluation and Management) study demonstrated that clinical practice guidelines could be implemented in Canada’s First Nation’s Communities leading to improved BP control. This community-based participatory randomized controlled research study in a Northern Canadian First Nations community was conducted in response to the community’s goal of controlling BP in people with diabetes to prevent the progression of kidney disease [20]. The hypothesis tested was whether the implementation of a chronic care model implemented by the addition of a home and community care nurse to support the chronic care model could provide a more effective means of lowering BP than usual care. Our research showed that the implementation of the new model led to significantly better BP control [20]. Two years after the completion of the DREAM3 study, BP in the treatment arm remained controlled, and BP in the control arm had fallen equal to that of the treatment arm [21].

The DREAM3 program included many of the components of a sustainable chronic disease intervention initiative, which are as follows: (1) patient self-management and participatory care; (2) decision support tools and easy-to-use, evidence-based aids for providers; (3) clarity of delivery team roles with planned coordination; (4) clinical information system that can track the progress of chronic disease management; (5) organization of health care delivery to accommodate the needs of chronic disease management; and (6) involvement of community-wide partnerships [22]. Furthermore, there was a focus on refreshing health education of skilled staff and educating new health care providers about new processes. The insights from the DREAM3 program have been integrated in the Dream-Global study, with an emphasis on changes in health services delivery supported by technology. Dream-Global incorporates patient self-management and participatory care by using texting (short message service, SMS) with BP readings, which provide the patient with the information they need to understand and participate in their own management. In the Dream-Global study, rather than increasing the burden of the home and community care nurses, task shifting was used to optimize the efficiency of the health care team and a dashboard was developed to track progress for each community. The SMS messages helped to link members of the interprofessional team, including the participant, the community health resource (CHR), the home and community nurse, and the participant’s primary health care provider.

SMS text messaging has become more widespread in research since the Dream-Global protocol was developed. A recent meta-analysis has demonstrated that text messages increased adherence to drug therapy [23]. Most studies compare the use of SMS messages to usual care; the Dream-Global study is unique in comparing disease-specific active messages to passive messages alone and their impact on BP control.

The research program described in the Dream-Global protocol focuses on high BP prevention and control in First Nations communities in Canada and rural communities in Tanzania. It builds on our research as well as previous research on the efficacy and effectiveness of approaches to prevention and control of high BP. The program is designed to work within existing health systems and workflows [24]. The research program involves the active engagement of the participating communities. It is designed to be adaptive to the settings where the research is occurring; factors to be taken into consideration include social, economic, and cultural contexts; disparities in population health and disease burdens; existing health care models for chronic disease management; level of awareness of the importance of hypertension as a disease burden; and existence of national clinical practice guidelines designed to reduce the burden of hypertension. In Canada, Indigenous populations include First Nations, Métis, and Inuit people. The study recruited participants from six First Nations communities, all of whom lived on reserve. In Tanzania, this program is being implemented in rural communities in Hai district of the Kilimanjaro region. Due to differences in the structure and financing of the health care system in both countries, the protocols for each country vary in their design and implementation. The SMS intervention remains the same but in different languages (English in Canada and Swahili in Tanzania) [25].

The implementation of a complex intervention such as the Dream-Global program is strongly affected by cross-cultural relationships between local partners and outside researchers. Trickett and colleagues have argued that “culture pervades all aspects of community interventions” [26]. Strong community engagement and community-based participatory research (CBPR) are the necessary foundation for successful implementation of a pragmatic trial, particularly in diverse geographic and cultural contexts [24]. The CBPR approach developed for Dream-Global is designed to incorporate local knowledge in the research process by meaningfully engaging those affected by the issue under study in all aspects of the research. This research program is also informed by the Behavioral Change Wheel (BCW), an evidence-based theoretical model that explains the individual and system changes necessary to support hypertension management in low-resource settings [27]. The BCW model incorporates essential internal and external conditions necessary for behavioral change, including changes in supporting policies, mechanisms, and systems, and patients’ capability, opportunity, and motivation [27]. The long-term goals of the Dream-Global research program are listed in Textbox 1. The program is designed to engage local decision makers and community leaders to involve multiple stakeholders from a variety of sectors, including the food industry, nursing, medical health, and public health, and to function smoothly with existing community programs. The components of the intervention will be robustly evaluated both individually and together to understand the relative contribution of each component and the synergies between them.

### Objectives

In this paper, we outline the methods, tools, and processes of the Dream-Global research program. This research program was designed to integrate innovations in communications technology and health services delivery with the local realities of health care while taking into account each community’s unique circumstances regarding geography, the social determinants of health, and the interplay between local, provincial, and federal health care systems.

This study includes the development of a community readiness to participate in research tool, a project to understand the sources of dietary sodium and how to reduce it in a remote community, the development of mobile health technology to incorporate BP measurement by nonmedical health workers, the transmission of the measurement to health care providers and patients, the development of guidelines-based culturally appropriate SMS messages for BP management, and the testing of these messages for BP lowering in a multicommunity randomized controlled trial in both Canada and Tanzania. The background information is provided here for both parts of the study, but for pragmatic reasons, the Tanzanian portion of the study was delayed and a fuller protocol is being prepared for separate publication.

**Dream-Global objectives.**

- To ultimately reduce the prevalence of cardiovascular disease, including heart attack and stroke, as a consequence of blood pressure lowering
- To enable scale-up of larger programs at the local, regional, and national levels
- To develop a better understanding of key barriers at local and national levels that affect hypertension control and how they can be overcome
- To understand how innovations for hypertension control can be introduced and scaled up
- To improve hypertension control rates, particularly in vulnerable populations

## Methods

### Study Goals

The purpose of this study is to investigate the efficacy of an evidence-based system of SMS messages linked to accurately measured BP in the community. The individual BP measurements are taken by community health workers (CHWs). This innovation allows for transmission of accurate BP measurements from patients in isolated or underserviced populations to health care providers and, subsequently, improvement of patient knowledge about hypertension management. Participants in the Dream-Global study are randomized to receive either SMS messages that are both active and passive, compared with those receiving only passive messages. All messages are derived from the Hypertension Canada Clinical Practice Guidelines and are subsequently modified with community input to make them culturally sensitive and specific in both Canada and Tanzania [25]. The 12 active messages explain the importance of BP control, describe the rational for medical therapy, and, based on the BP reading, may instruct the participant to contact their health care provider for further BP follow-up. The 26 passive messages include health behaviors change advice for diet and exercise. Messages are sent twice weekly, on Monday and Thursday at 11 AM (to avoid holidays). The study is divided into five projects that involve the design, implementation, and then staging of the research program for the successful execution of a randomized controlled trial in both Canada and Tanzania.

*Project 1* is to develop a readiness to participate tool. The tool entails a multidimensional checklist intended to assess community readiness for implementation of a research program for hypertension awareness, treatment, and control. The tool developed was piloted in both Canadian Indigenous and Tanzanian communities participating in the Dream-Global study with evaluation and feedback for validation [24].

*Project 2* is a substudy to determine whether the sodium consumption of Indigenous people can be modified to help prevent hypertension. The goal was to plan and implement a food procurement policy for reducing sodium and fat intake from commercial food sources in an isolated Northern Indigenous community. This was approached by collating scientific evidence and existing policy recommendations, engaging community leaders and local food suppliers, and developing a policy approach for commercial food procurement practices. Evaluation includes process measures and examination of successes and barriers to implementation.

*Project 3* entails the development of a closed loop app to transmit BP results taken by the CHR in Canada and the CHW in Tanzania to the health care provider and to the participant in a manner consistent with personal health privacy rules in each country.

*Project 4* includes the development of a guidelines-based set of SMS messages in English that are culturally appropriate for Canadian First Nations communities and a set of SMS messages in Swahili that are culturally specific and appropriate for rural Tanzanian communities.

*Project 5* will evaluate the impact of the active and passive messages together, compared with that of passive messages alone on BP control over 1 year through a prospective, pragmatic, randomized controlled trial in both Canada and Tanzania.

### Blood Pressure Screening: Canada

In the Canadian Indigenous communities, the community nurse and CHR, a nonmedical health worker (analogous to CHWs in Tanzania), was trained in all aspects of the study, including BP measurement with the automated device, how to recruit, and how to consent participants. Consent was obtained from all participants, including the CHR, community nurse, and other members of the health care team and Band leadership who participated in surveys and interviews, as well as all participants for the BP measurement and lowering study.

Community leadership recommended that all interested individuals be allowed to participate as long as they are eligible. The study initially had focused on BP lowering, but to meet the community needs, a BP finding substudy was expanded to allow participants who were medically untreated but who screened below enrollment thresholds to participate. The screening BP was measured by trained Home and Community Care nurses or CHRs in community clinics using the BpTRU (BpTRU Medical Devices Ltd, Coquitlam, British Columbia, Canada), which takes six readings, omitting the first and averaging the final five. This BP reading, along with the participant’s baseline demographics, was recorded on a data collection form, which was deidentified and faxed back to the study center. Participants for the BP lowering study had to have uncontrolled hypertension while being on or off medications. Uncontrolled hypertension was defined as 140/90 mm Hg or above, or 130/80 mm Hg or above for participants with diabetes. In this way, only people on medications and who had BP controlled to target were not eligible. By limiting subjects who were ineligible for ongoing BP monitoring and SMS messages, the study met the needs of the community. It was recognized that some subjects who appeared to have controlled BP at screening might turn out to be uncontrolled and that some who appeared to be uncontrolled at screening might later turn out to be controlled.

Eligible participants were registered online. At registration, they were randomized electronically to receive active and passive SMS messages or passive only, and their names were then listed on the Dream-Global mobile app on the smartphone used by the Home and Community Care nurse or CHR in their community. BP was measured with the A&D UA-767PBT-C monitor (A&D Medical, San Jose, CA) to test that it was being transmitted properly; an SMS message to the participant’s registered mobile phone, with the BP reading, was the confirmation of transmission. The Home and Community Care nurse and CHR were trained to take three BP readings with this device. The study server was programmed in such a manner that it waits for up to three readings and then sends their mean to the participant’s own phone. Randomization through the study server ensured that participants and study personnel were blind to the allocation.

The baseline BP was defined as the mean of all readings from the A&D device in the first 2 months after randomization. If the average BP was uncontrolled during the first 2 months, the patient was identified as being in the BP lowering study; otherwise, they were followed up for BP screening. BP readings were stored on the central server, and deidentified data were transferred monthly to the data analysis center in Ottawa.

### Blood Pressure Screening: Tanzania

A 6-week BP screening exercise in Tanzania was used as training to test the technology and the operationalization and feasibility of the protocol implementation in the participating rural communities in Kilimanjaro, Tanzania. CHWs were trained to utilize an Android smartphone with the study app. All eligible participating members were invited to participate in the BP screening study to measure community interest.

### Canada-Specific Considerations

The Canadian arm of the project was carried out in six Indigenous communities located in three different geographic regions, including Ontario’s Manitoulin Island, the James Bay Coast of Quebec, and the north shore of New Brunswick. Recruitment into the study began in the spring of 2013 and was completed by November 2015. The last patient exited the study at the end of 2016. Study participants were followed for a period of at least 1 year. Communities were selected through a semirandom method. Canadian Indigenous communities can be found on an interactive map maintained by the Government of Canada with profiles, including population information [28]. Potential communities included those with a population of at least 3000, existing 3G mobile phone service, stable governance, and a clinical champion to serve as a bridge between the Dream-Global study researchers and the community. Potential communities were listed and put into a random order. They were contacted one by one until it was felt that sufficient communities had been contacted to make the planned study enrollment. Communities were later assessed with the Intervention and Research Readiness Engagement and Assessment of Community Health Care (iRREACH) tool (see below) to determine their readiness for research and the necessary study adaptations required to meet their needs [24].

Following an invitation by the community to discuss the study, a visit was arranged with the Canadian study lead (ST) and study staff (NP) to introduce the Dream-Global research program. This was followed by another visit to determine research readiness with the iRREACH community research participation tool [24]. A Band or Tribal Council resolution was required to ensure that the study was endorsed by the community leadership. This was particularly important because the nonmedical worker, or CHR, was central to the functioning of the study. The CHR was not compensated by the study but rather by the community who felt that the study was of sufficient importance, including research capacity development to provide enough of the CHR’s time to manage hypertension (typically 1 day per week).

The CHRs participating in the study in each community were identified by the community leadership. The CHR worked closely with a Home and Community Care nurse, and both were trained by the Dream-Global study staff to perform the BP measurements, to recruit, consent, and enroll study participants. The training took place over a period of 2 days and was conducted by one of the Dream-Global staff (NP). The training manual was developed with input from the communities and was designed to be culturally safe. As needed, weekly teleconferences were held between the CHRs, the Home and Community Care nurse, and the Dream-Global study team. These sessions were used to troubleshoot challenges with the technology, recruitment strategies, and BP management. The CHR or the Home and Community Care nurse were aware of the BP readings but were instructed not to act on them unless the readings were 180/100 mm Hg or higher or the participant appeared to be in distress. Every effort was made to get medical attention for the participant on an escalating scale based on the BP reading through the local health care system, even to the point of activating the local emergency medical system. Study staff were available at all times through their personal mobile phones via SMS texting or direct calls. Focus groups and key informant interviews were undertaken at the beginning and end of the study to understand the health team’s response to the Dream-Global technology. Exit interviews were arranged for participants, including a knowledge test of the content of the SMS messages.

### Pragmatic Aspects of the Randomized Controlled Trials

Pragmatic randomized controlled trials (RCTs) are designed to evaluate an intervention in real-life routine practice conditions in contrast to traditional explanatory RCTs that operate in optimal conditions [29]. When working with Indigenous community–based health research, including community engagement and community involvement in the design and study implementation, a pragmatic RCT supports data collection in a respectful and participatory way. It can determine whether the intervention is effective and works in a real-life setting. The Dream-Global study was designed to be a pragmatic study to meet the needs of the participating communities, to provide the ability to do a process evaluation to understand the ultimate study results, and to increase the probability that evidence from the study could be used in clinical practice and policy [30].

### Eligibility Criteria

#### Inclusion Criteria

Inclusion criteria were as follows: age 18 years or older and a diagnosis of hypertension for at least 12 weeks before screening. If already on drug therapy, continuation of the same dosing interval for 8 weeks before the screening for BP. Participants must be able and willing to provide written informed consent, possess a mobile phone capable of receiving SMS text messages, or, for those in Canada with no phone, willing to carry and learn to use a flip phone for the study duration.

#### Exclusion Criteria

People who meet the eligibility criteria for either of the above studies but have poorly controlled hypertension (BP>180/110 mm Hg), have an active malignant disease (except nonmelanoma skin cancer), are planning elective surgery during the study period (except for cataracts), are participating in another clinical trial, do not have a primary health care practitioner, are unable or unwilling to visit a health care provider, and/or are unable to read the SMS messages are ineligible for study participation. People on investigational drugs (in the 3 months before initial screening visit or during screening and treatment periods) or using SMS-related interventions (in the 12 weeks before screening or during screening and treatment periods) are also ineligible.

### Local Health Provider Engagement and Support

In Canada, a continuing professional development information session was made available for local primary health care practitioners on the Dream-Global research program and hypertension clinical practice guidelines were updated. These sessions were held at least once for each region and took place on reserve or close to the reserve. In addition to the extensive training on the Tanzanian Standard Treatment Guidelines for Hypertension (2013) provided to the local health providers at the participating health centers, the research team in Tanzania collaborated with a local referral hospital to host an event on updated hypertension treatment guidelines and cardiovascular disease management.

### Sample Size

For BP lowering, a reduction in systolic BP of 5 mm Hg is associated with a 14% reduction of stroke, a 9% reduction of coronary heart disease mortality, and a 7% reduction in total mortality; even reductions of 2 mm Hg are associated with significant benefits (−6%, −4%, and −3%, respectively) [28]. Thus, a significantly important difference could be as low as a 2-mm Hg change in systolic BP. In the DREAM3 study groups, systolic BP changed by mean 24.0 (SD 13.5) mm Hg and 17.0 (SD 18.6) mm Hg, with a between-groups difference of 7 mm Hg. Using the more conservative change in diastolic BP from that study, with mean 11.6 (SD 10.6) mm Hg versus 6.8 (SD 11) mm Hg in the control group (mean difference 4.8 mm Hg), provides an intraclass correlation coefficient for BPs between groups of 0.0133. This translates to an inflation factor of 1.1729 with 176 subjects per group for power of 97% to find the difference. Thus, a target was set for 360 participants to be recruited. The extra power was built in to allow for underrecruitment and a smaller difference in BPs between the groups.

The BP finding study is run as a pilot to determine the role of the Dream-Global program to identify the new onset of hypertension.

### Blinding and Randomization

Participants were randomly allocated after enrollment using random number sequence with blocking of 2s and 4s for each community so that participants and study staff would not know which group participants had been enrolled. The randomization to receive active and passive or passive only text messages took place at the time of enrollment by the central server software. This information was available only to the database manager; participants, clinicians, and study staff had no knowledge of randomization.

### Participant Flow

Canadian community members were invited to participate in the study through local advertisements and community engagement (town hall meetings and messages from the Band leadership). Referrals were also expected to come from all community health practitioners and Band leadership.

After enrollment, all BPs are measured with the A&D Bluetooth-enabled BP device by the CHR or Home and Community Care nurse in Canada or the CHW in Tanzania. Canadian participants typically meet the CHR in their office in the Band Council’s health facility on reserve. In Canada, all consent forms were in English but could be translated for participants by the CHR if necessary. In Tanzania, all study information and consent forms are pretranslated into Swahili.

Attempts were made to discourage people living in the same house from enrolling in the study to reduce the potential for cross contamination between the messages. However, to respect the community leadership in both countries to make participation widely available, this could be overruled by the CHRs and CHWs if they felt it was necessary.

### Generalizability

The study took place in Indigenous reserves in Canada and two rural communities in Tanzania. These communities were rural and remote, had limited access to primary care providers, and, in Tanzania, often had difficulty accessing medication for treatment continuity. In these communities, inconsistent BP measurement and follow-up, as well as poor access to antihypertensive medications, were known to contribute to poorer rates of BP control.

### Statistical Analysis

All patients who receive at least one SMS message and for whom data for one or more follow-up BP reading is available will be included in the efficacy and safety analyses.

### Efficacy Analyses

The primary efficacy endpoint in this study will be assessed by analysis of variance. Descriptive data will be given, with the mean and standard deviation for continuous data and proportions for ordinal data. Preplanned exploratory subgroup analyses will include analysis by community, sex, diabetes status, phone ownership at baseline, and age. The knowledge gained from the text messages will be assessed by questionnaire at study completion, and results will be compared between the groups.

### Safety Analyses

The primary study safety variable is the overall incidence of adverse events (AEs) between study entry and end. Descriptive summary statistics will be presented for the number and severity of AEs reported by each patient, overall and by individual organ system. The upper limit for the 95% one-sided CI for the incidence of the most frequent AE will be calculated.

### Meta-Analysis

This study is one of the series of clinical trials being undertaken by the Global Alliance on Chronic Disease (GACD). In addition to the study-specific analyses outlined above, study data will become part of a common database for all clinical trials in the GACD hypertension series to perform meta-analyses. These analyses will be defined prospectively and are intended to identify subpopulations and other baseline characteristics of treatment patients that may influence treatment outcomes. Only denominated data will be used. Analysis and dissemination will take into account the principles of shared ownership of research findings held by university-based researchers and community partners as outlined in the research agreements.

### Development of SMS Message Bank

The development of the SMS messages took place through a process of community engagement with Indigenous and Tanzanian communities who were motivated to participate in the research program. Through a series of focus groups and individual interviews with health care providers and people with hypertension, as well as with other community stakeholders, we developed culturally appropriate and acceptable SMS messages through a process that has been published [26].

### Quality Control

The overall procedures for quality assurance of clinical study data follow those of Good Clinical Practice and the principles of ethical research involving the First Nations, Inuit, and Métis People of Canada as put forth in the Tri-Council Policy Statement 2 (TCPS2) [27]. A data safety monitoring board was established for this study to monitor the study annually.

### Ethics Approval

Approval to conduct the study in Canadian and Tanzanian communities was granted by community leadership in each community. Furthermore, in Tanzania, approval consent and advice was also sought from the Kilimanjaro Regional Medical Officer (RMO), the Hai District Medical Officer (DMO), and the National Institute of Medical Research (NIMR), whose ethics board reviewed the protocol.

The study protocol has been approved by local ethics boards, including The Cree Board of Health and Social Services of James Bay, Ontario (July 10, 2013); the Cree Nation of Chisasibi (August 15, 2013); Manitoulin Anishnabek Research Review Committee (October 7, 2013); the National Institute for Medical Research in Dar es Salaam, Tanzania (approved on March 19, 2014); and Northwest Territories Scientific Research License number 15317 (August 8, 2013). It has also been approved by institutional ethics boards, including the University of Calgary (REB13-0573; September 23, 2013); Queen’s University Health Sciences and Affiliated Teaching Hospitals Research Ethics Board, Kingston, Ontario (DMED-1603-13 approved on June 21, 2013); Sunnybrook Research Institute Research Ethics Board, Toronto, Ontario (ID number 053-2013; May 10, 2013); and the National Institute for Medical Research Tanzania (NIMR/HQ/R.8a/Vol.IX/1698).

### Privacy and Cultural Considerations

The study data are housed in a secure central server with appropriate safeguards for data security and backup and appropriate privacy provisions to avoid deidentification of personal data. The study will maintain the principles of Good Clinical Practice and the principles enunciated in the Declaration of Helsinki and the TCPS on ethical conduct for research involving humans [31,32]. This study will adhere to the principles of ownership, control, access, and possession; our group has experience of working within this important framework [33]. The partners already adhere to the principles of the Personal Information Protection and Electronic Documents Act (PIPEDA), and movement of data in this study will be compliant with this act.

### Program Governance

The Dream-Global team was designed to be multidisciplinary and interprofessional, including First Nations community representatives. The research program brings together nationally recognized leaders in clinical practice guidelines’ implementation and clinical trials and research in hypertension, with expertise to work collaboratively with First Nations communities. Decision makers and the multidisciplinary team members were invited to provide letters of support to clarify their roles. A steering committee was developed and tasked with ensuring that proposed intervention strategies were appropriate for the social, cultural, and economic contexts. Annual research meetings provided a forum to share plans with broader team and community members, as well as to receive feedback before implementing plans. Research reports were also developed and presented to the annual GACD research forum, which included oversight from the CIHR.

### Knowledge Translation and Engagement

Annual presentations will be made to each community on the study’s progress. At the end of the study, presentations are made to the community leadership, health care providers and participants, and community members through town-hall style meetings. Results will also be summarized in a poster for the communities. Annual presentations are made at the GACD study meeting, at which the other 14 study teams also present their updates.

**Requirements of the Dream-Global app.**

- Participants must be registered securely.
- Participants’ names will only show up on their community health resource’s or community health worker’s Dream-Global smartphone.
- Blood pressure readings on the A&D Medical monitor would transmit by Bluetooth to the smartphone.
- Blood pressure readings on the smartphone would be transmitted to the central server.
- A mean of the readings received by the central server would then be sent to the participant’s own phone (different from the community health worker’s smartphone).
- The central server will send the mean of the readings to the participant’s health care provider by fax in the form of a letter, based on the information provided at registration (Canada only).
- The server will be programmed to assess the blood pressure reading as normal or high and, if high for the participants receiving active messages, to advise them to see their health care provider for blood pressure management. In Canada, messages will include the phone number of their health care provider; in Tanzania, messages will include the location and weekdays on which the local blood pressure assessment clinic is running.

Abstracts will be presented at local, national, and international meetings as appropriate, and there will be publications in the peer-reviewed literature documenting the study progress and results. Furthermore, semiannual presentations will be made to Health Canada, specifically for the First Nations and Inuit Health Branch that funds the Home and Community Care health delivery in most First Nations communities. Semiannual progress reports are also submitted to the National Institute for Medical Research Tanzania. 

Other efforts will be made to present to policy makers and payers and to community leadership to promote sustainability and scalability at the end of the study. Interested parties will be given the chance to read (or have read to them) the information sheet and ask any questions before providing written consent. Copies of the information sheet and consent form will be given to study participants. All personally identifying information will be removed from study documents. Study participants will be identified using a unique alphanumeric number. To track participants, a list linking the name and home address of each person will be maintained in a locked filing cabinet.

### Technical Aspects of mHealth Component

The app and database are hosted at a Health Insurance Portability and Accountability (HIPPA)-compliant Canadian Provincial Crown Corporation providing sufficient reliability to maintain 99.999% uptime to meet the study needs. The field interface includes automated BP measurements by CHWs trained to do BP measurements according to the guidelines. The Bluetooth-enabled automated BP device (A&D Medical, UA-767PBT-C, San Jose, California) then transmits information via Bluetooth to the Android or BlackBerry mobile device carried by the CHW. The requirements of the app are listed in Textbox 2. The tools were tested and validated in pilot focus groups in participating communities.

The messages will be sent via a central app and database server that is ISO1348 and Food and Drug Administration–certified and brokered by an International SMS Gateway to study participants. To facilitate the BP measurement and interaction between patient and provider, algorithms were developed both at the app server level (in the cloud) and on Android/Blackberry-based devices for deployment in the field.

### Blood Pressure Control Study and Primary and Secondary Outcomes

For the BP control study, the primary outcome is the difference between the two groups in BP change from baseline to study end (both systolic and diastolic). One year was chosen as a target for BP monitoring to allow for sufficient measurements and to reduce the Hawthorne effect, in which improved BP control from better adherence might occur for the period after study enrolment [34]. To reduce the effect of regression to the mean, the baseline BP was defined as the mean of the BP readings (each mean of three measures) taken with the A&D UA-767PBT-C in the first 2 months after enrolling in the study. The final BP measurement is the mean of the last 2 to 3 measurement days, usually in the last 2 months, to reduce variability from a single set of readings. The secondary outcome is the proportion of participants achieving BP control during the study period.

## Results

The iRREACH tool was developed and used in all study sites in both countries [24]. Text messages derived from the Hypertension Canada Clinical Practice Guidelines were developed and revised based on focus groups in both countries as has been described [26]. A process evaluation is being developed [35]. Six communities were enrolled in the study in Canada, and recruitment was completed in November 2015. The last patient completed the study in December 2016. In total, 234 participants enrolled in the program and had at least one set of transmitted BP readings.

## Discussion

The purpose of this research program is to integrate technologic solutions with innovations in health service delivery to improve the treatment and control of hypertension. This requires integration of the scientific and social aspects of this study with business and governmental partners to design proper public-private partnerships that can be sustainable over time. Users and health care providers are involved in the development and refinement of this technology from its outset to ensure that it is useful for them. The data gathered will hopefully be of great interest to businesses and policy makers for developing ongoing sustainable business models and for scaling up in more communities. It will also inform community decision makers and governmental partners so they can promote policy changes. One marker of success for this project will be obtaining funding from Health Canada to maintain the program for the communities participating in Dream-Global, as well as others who become interested in participating at study end.

In conclusion, this study is expected to provide useful insights into the acceptability and effectiveness of an innovative system of community-based screening, treatment, and follow-up for hypertension in remote Indigenous communities in Canada. If it proves successful in this environment, then it should also be beneficial in nonindigenous settings in rural and remote areas, as well as urban settings. This work will expand an already successful body of research on bringing clinical evidence into daily practice for hypertension and can serve as a model for addressing other noncommunicable diseases in settings with limited access to health care resources. Integrating innovations in technology, health service delivery, and business models is a novel potential approach to achieve sustainable control and prevention of hypertension in particular and to address the *implementation gap* in chronic diseases in general. We will demonstrate whether the simple yet practical solution of linking hypertensive individuals with their health care providers and their personal health and medical records through SMS technology on mobile phones will lead to improved BP control and, by extension, improved outcomes.

## Appendix

Multimedia Appendix 1 CIHR peer-review report.

## Abbreviations

AE: adverse events
BCW: Behavioral Change Wheel
BP: blood pressure
CBPR: community-based participatory research
CHR: community health resource
CHW: community health workers
DMO: district medical officer
DREAM3: Diabetes Risk Evaluation and Management
GACD: Global Alliance on Chronic Disease
iRREACH: Intervention and Research Readiness Engagement and Assessment of Community Health Care
LMIC: lower- and middle-income country
NIMR: National Institute of Medical Research
RCT: randomized controlled trial
RMO: regional medical officer
SMS: short message service
TCPS2: Tri-Council Policy Statement 2
