# Supplementary material for: The Effectiveness of Text Messaging for Detection and Management of Hypertension in Indigenous People in Canada: Protocol for a Randomized Controlled Trial
Source: JMIR Res Protoc. 2017 Dec 19;6(12):e244. doi: 10.2196/resprot.7139 (PMC5750415; doi:10.2196/resprot.7139)
Supplement: Multimedia Appendix 1 [file resprot_v6i12e244_app1.pdf]

March/Mars 01, 2012

DC0190GP

Canadian Institutes of Health Research / Instituts de recherche en santé du Canada

Notice of Decision / Avis de décision

Application Number/Numéro de la demande: 260941

Committee Code/Code du comité: IHR

Applicants/Candidats: Dr. Peter P. LIU, Dr. Norman Rc CAMPBELL, Dr. Sheldon William TOBE, Dr. Karen YEATES

With/Avec: Dr. O. BHATTACHARYYA, Dr. K. KILONZO, Mrs. M. MOY LUM-KWONG, Ms. J. WENTWORTH

Institution paid/ Établissement payé: University Health Network (Toronto)

Title/Titre: DREAM-GLOBAL: Diagnosing hypeRtension - Engaging Action and Management in Getting LOwer Bp in Aboriginal and LMIC - A Research Proposal

Primary Inst./ Inst. principal: Circulatory and Respiratory Health

Other Related Inst./ Autres inst. connexes: Aboriginal Peoples' Health; Health Services and Policy Research; Population and Public Health

Competition Outcome/Résultats du concours: Team Grant: Implem Res on Hypertension in Low/Middle Income Countries
September/Septembre 01, 2011

Number in competition/Nbre de demandes dans le concours: 12

Number approved/Nbre de demandes approuvées: 3

Decision on your application/ Décision sur votre demande: Approved

Average annual amount/ Montant annuel moyen: $360,820

Equipment amount/ Montant pour les appareils: $0

Term/Durée: **5 yrs/ans 0 months/mois**

Peer Review Committee Recommendation, for your information and use/ Recommandation du comité d'examen par les pairs, pour fins d'information et d'utilisation:

Committee/Comité: Implementation Research on Hypertension in LMICs

Application rank within the competition/ Rang de la demande dans ce concours: 3

Percent Rank Within the Competition/ Rang en pourcentage au sein du concours: 25%

Rating/ Cote:
- Potential Impact: 4.20
- Scientific Merit: 4.08

Recommended average annual amount/ Montant annuel moyen recommandé: $397,432

Recommended equipment amount/ Montant recommandé pour les appareils: $1,800

*** Applications receiving a score of less than 3.5 on any evaluation criteria will not be considered for Funding. / Les demandes qui ont reçu une note inférieure à 3.5 pour n'importe quel des critères d'évaluation ne sont pas admissibles.

This document is for information only. Official payment is stated on the CIHR Authorization for Funding.
Document à titre d'information seulement. Le paiement officiel est indiqué sur l'autorisation de financement des IRSC.
